# A Systematic Review of the Risk of Non-alcoholic Fatty Liver Disease in Women With Polycystic Ovary Syndrome

**DOI:** 10.7759/cureus.29928

**Published:** 2022-10-04

**Authors:** Mahrukh Shahbaz, Halah Almatooq, Paul Foucambert, Faith D Esbrand, Sana Zafar, Venkatesh Panthangi, Adrienne R Cyril Kurupp, Anjumol Raju, Gaurav Luthra, Safeera Khan

**Affiliations:** 1 Internal Medicine, California Institute of Behavioral Neurosciences & Psychology, Fairfield, USA; 2 Dermatology, California Institute of Behavioral Neurosciences & Psychology, Fairfield, USA; 3 Pediatrics, California Institute of Behavioral Neurosciences & Psychology, Fairfield, USA

**Keywords:** non-alcoholic steatohepatitis (nash), endocrine disorders, women of reproductive age, nonalcoholic fatty liver disease (nafld), polycystic ovary syndrome (pcos)

## Abstract

Polycystic ovary syndrome (PCOS) is a complex hormonal disorder associated with complications throughout various body organs. Previous studies have shown evidence of liver disease in some women with PCOS. In this study, we attempted to explore the risk of non-alcoholic fatty liver disease (NAFLD) in PCOS women and the specific factors involved in its development. We searched PubMed, PubMed Central, Medline, and ScienceDirect for articles related to the topic, screened those articles according to our inclusion/exclusion criteria, and conducted a thorough quality check using various quality appraisal tools to select articles relevant to our research. The process was conducted according to Preferred Reporting Items for Systematic Review and Meta-Analyses (PRISMA) Checklist 2020.

We selected 11 high-quality observational studies for our review. Studies from various countries were included, and all studies demonstrated an increased prevalence of NAFLD in PCOS patients compared to healthy controls. Although insulin resistance, obesity, and increased androgens contribute to the increase in the risk of NAFLD in these patients, hyperandrogenism was the most influential risk factor in four of these studies. Two studies explored the degree of NAFLD in these patients using transient elastography (TE). They concluded that PCOS was significantly associated with hepatic steatosis (HS) rather than hepatic fibrosis in most patients. PCOS patients have an increased risk of developing NAFLD, particularly HS, and hyperandrogenism seems to be the main determinant. Therefore, effort should be put into screening and monitoring these patients to manage the disease. TE may be a useful method for monitoring the natural history of NAFLD in these patients, which requires further exploration.

## Introduction and background

Polycystic ovary syndrome (PCOS) is an endocrine disorder that affects one in 10 women of reproductive age [1]. In these women, the ovaries produce excessive androgens leading to hormonal imbalance. This disorder is characterized by hyperandrogenism, polycystic ovaries, and anovulation. While the exact cause of PCOS is not known, experts have linked it to genetics, hyperandrogenism, insulin resistance (IR), and low-grade inflammation [2]. Its diagnosis requires a complete medical history, a physical exam, pelvic exam, pelvic ultrasound, and blood tests [1]. The treatment varies greatly but may include metformin, clomiphene citrate, letrazole, aromatase inhibitors, laparoscopic ovarian drilling, and gonadotropins [3]. Studies on the efficacy of such treatments are ongoing as there is no known cure for the disease. PCOS has been linked to many complications, including infertility, metabolic syndrome (MetS), cardiovascular disease, obstetric cancers, and psychological disorders [3]. In addition, many studies have tried to show a link between PCOS and non-alcoholic fatty liver disease (NAFLD), undoubtedly because the risk factors of NAFLD are also co-morbidities found in PCOS [4].

NAFLD is the fat accumulation in the liver independent of alcohol consumption greater than 5% to 10% of the liver's total weight [5]. Fat accumulation results in an inflammatory response that damages the parenchyma and may progress to non-alcoholic steatohepatitis (NASH), cirrhosis, and eventually liver failure or cancer [6]. NAFLD is called a silent disease because it is largely asymptomatic. Diagnosis of the condition involves a medical history, physical exam, imaging studies like ultrasound and CT scan, and lastly, liver biopsy [7]. Treatment is yet to be established, but it can be managed by changing to a healthier lifestyle.

It is well-known that people with obesity, MetS, and type 2 diabetes mellitus often develop NAFLD [7]. Thus, the early studies that established an association between PCOS and NAFLD attributed the association to MetS, which can be present in patients with PCOS and those with NAFLD. Studies regarding the pathogenesis of NAFLD in PCOS patients are inconsistent and inconclusive. Thus, an updated systematic review was conducted to better understand the risk of NAFLD in women of reproductive age with PCOS.

## Review

Methods

This systematic review was conducted and reported according to the Preferred Reporting Items for Systematic Review and Meta-Analyses (PRISMA) 2020 checklist [8].

Search Sources and Search Strategy

We used PubMed, PubMed Central, Medline, and ScienceDirect to retrieve relevant articles. "Polycystic Ovary Syndrome" and "Non-alcoholic Fatty Liver disease" were the keywords used in PubMed and the other databases with the boolean AND. Medical subject heading (MeSH) strategy was employed in PubMed in addition to keyword search, which was as follows: ("Polycystic Ovary Syndrome/complications"[Mesh]) AND "Non-alcoholic Fatty Liver Disease"[Mesh]. This way, we could identify several articles that linked PCOS with NAFLD.

Eligibility Criteria

Strict inclusion criteria were followed to select good-quality articles for this systematic review. These include the following: (i) any papers focusing on girls or women of reproductive age; (ii) papers written in the English language; and (iii) papers relevant to the research question.

Conversely, the exclusion criteria involved (i) any papers discussing post-menopausal women; (ii) papers published as Grey Literature; (iii) any papers without an abstract; (iv) papers discussing animal studies; (v) any unpublished literature; and (vi) any studies without method section.

Screening

The screening process began with filtering out a hefty number of articles on ScienceDirect. Any review articles, research articles, and articles under medicine and dentistry were selected. This was followed by a compilation of a list of articles from each database in Microsoft Excel. Any duplicates were then removed. Next, articles with titles and abstracts pertinent to the topic were selected. Then, any articles for which full texts could not be accessed were excluded. Lastly, the inclusion and exclusion criteria were applied.

Results

Search Outcomes

**Figure 1:**
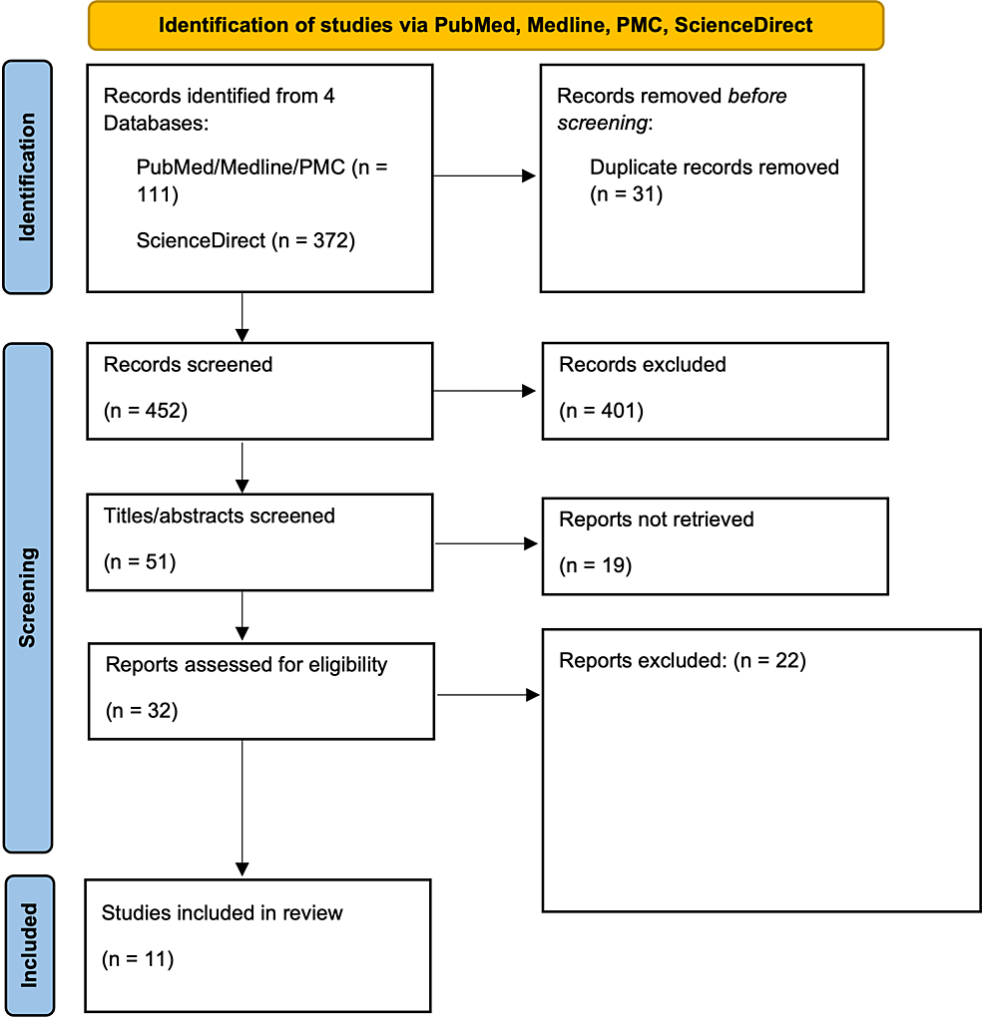
PRISMA 2020 flow diagram for systematic review PRISMA = Preferred Reporting Items for Systematic Review and Meta-Analyses; PMC = PubMed Central

We identified a total of 983 articles from the selected databases. Eight hundred seventy-two of these articles were from ScienceDirect, of which 500 were removed after the following filters were applied: review articles, research articles, medicine, and dentistry. This left us with a total of 483 articles. Thirty-one duplicates were then removed. The screening began by selecting articles with titles that were relevant to the question. This resulted in the removal of 365 articles. Next, 36 articles were removed due to a lack of access to abstracts or abstracts that were unrelated to the research question. Nineteen of the remaining 51 articles were removed because we could not access the full-text articles. Finally, 32 articles were screened via the chosen inclusion and exclusion criteria. Twenty-two articles underwent quality appraisal, leaving only 11 to be evaluated. The PRISMA flowchart in Figure 1 demonstrates the article selection process.

Results of Quality Appraisal

The remaining articles were then critically assessed using quality appraisal tools to develop a final list of high-quality, relevant articles to be reviewed. Most of the studies included in this review were observational studies for which the Joanna Briggs Institute (JBI) critical appraisal tool was used. Assessment of multiple systematic reviews (AMSTAR) checklist was used for a few systematic reviews and meta-analyses. This systematic review contains all articles that satisfied at least 70% of the criteria in each critical appraisal tool. Tables 1-4 show the results of the quality appraisal.

**Table 1:** JBI checklist for quality appraisal of case-control studies JBI = Joanna Briggs Institute

Case-control studies	Kim, JJ et al., 2017 [9]	Vassilatou et al., 2010 [10]	Zueff et al., 2012 [11]
Were the groups similar other than the presence of disease in cases or the absence of disease in controls?	Yes	Yes	Yes
Were cases and controls matched?	Yes	Yes	Yes
Were the same standards used for identification of cases and controls?	Yes	Yes	Yes
Was exposure determined in a standard, precise, and accurate way?	Yes	Yes	No
Was exposure measured the same way for both cases and controls?	Yes	Yes	Yes
Were confounding factors identified?	Yes	Yes	No
Were strategies to deal with confounding factors specified?	Yes	Yes	No
Were outcomes evaluated in a standard, valid and reliable way for cases and controls?	Yes	Yes	Unclear
Was the period of exposure long enough to be meaningful?	Yes	Unclear	Unclear
Was suitable statistical analysis used?	Yes	Yes	No

**Table 2:** JBI checklist for quality appraisal of cross-sectional studies JBI = Joanna Briggs Institute

Cross-section-al studies	Asfari et al., 2020 [4]	Salva-Pastor et al., 2020 [6]	Taranto et al., 2020 [12]	Polyzos et al., 2014 [13]	Chakraborty et al., 2020 [14]	Macut et al., 2016 [15]	Jones et al., 2012 [16]	Michaliszyn et al., 2013 [17]	Romanowski et al., 2015 [18]	Bohdanowicz-Pawlak et al., 2014 [19]
Were criteria for inclusion in the sample clearly described?	Yes	Yes	Yes	Yes	Yes	Yes	Yes	Yes	Yes	Yes
Were the study subjects and the setting explained in detail?	Yes	Yes	Yes	Yes	Yes	Yes	Unclear	Yes	Yes	No
Was the exposure assessed in a precise and accurate method?	Unclear	Yes	Yes	Yes	Yes	Unclear	Yes	Unclear	Unclear	Yes
Were objective and standard criteria used for measurement of the condition?	Yes	Unclear	Yes	Yes	Yes	Yes	Yes	Unclear	Yes	Yes
Were confounding factors acknowledged?	Yes	Yes	No	Unclear	No	Unclear	Yes	No	No	No
Were strategies to deal with confounding factors specified?	Yes	Yes	No	Yes	No	Yes	Yes	No	No	No
Were outcomes assessed in a way that is accurate and precise?	Unclear	Yes	Yes	Unclear	Yes	Yes	Unclear	Unclear	Unclear	Yes
Was appropriate statistical analysis used?	Yes	Yes	Yes	Yes	Yes	Yes	Yes	Unclear	Unclear	Yes

**Table 3:** JBI checklist for quality appraisal of cohort studies JBI = Joanna Briggs Institute

Cohort studies	Won et al., 2021 [20]	Shengir et al., 2020 [21]	Kumarendran et al., 2018 [22]	Petta et al., 2017 [23]	Gambarin-Gelwan et al., 2007 [24]	Economou et al., 2009 [25]	Sarkar et al., 2020 [26]
Were the two groups similar and enlisted from the same population?	Yes	Yes	Yes	Yes	Yes	Yes	Unclear
Were the exposures measured comparably to assign people to exposed and unexposed groups?	Unclear	Yes	Yes	Yes	Yes	Yes	Yes
Was the exposure assessed in a method that was accurate and precise?	Unclear	Yes	Yes	Yes	Yes	Unclear	Yes
Were confounding factors acknowledged?	No	Yes	Yes	Yes	No	Yes	Yes
Were strategies to deal with confounding factors specified?	No	Yes	Yes	Yes	No	Yes	No
Were the participants outcome-free at the start of the study (or at the moment of exposure)?	Unclear	Yes	Yes	Unclear	Yes	Yes	No
Were outcomes assessed in a valid and reliable way?	Yes	Yes	Unclear	Yes	Yes	Unclear	Yes
Was the follow-up time reported sufficiently long for the outcome?	Yes	No	Yes	No	Yes	No	Yes
Was follow-up complete? If not, were the reasons for loss of follow-up explained?	Yes	Unclear	Yes	Unclear	Yes	Yes	Yes
Were strategies to address incomplete follow-up used?	No	No	No	No	No	No	No
Was appropriate statistical analysis utilized?	Yes	Yes	Yes	Yes	Unclear	Yes	Yes

**Table 4:** AMSTAR checklist for quality appraisal of systematic reviews AMSTAR = Assessment of multiple systematic reviews; PICO = patient/population, intervention, comparison and outcomes; RoB = risk of bias

AMSTAR Checklist	Baranova et al., 2011 [27]	Wu et al., 2018 [28]
1. Did the research questions and inclusion criteria include PICO?	No	Yes
2. Did the review contain a statement that the review methods were established before conducting the review, and did the report justify any important differences from protocol?	No	Yes
3. Did the review authors clarify their selection of the study designs for inclusion in the review?	No	Yes
4. Did the authors use a broad literature search strategy?	No	Yes
5. Did the authors perform the study selection in duplicate?	Yes	Yes
6. Did the authors extract data in duplicate?	Yes	Yes
7. Did the authors provide a list of studies that were not included and rationalize the exclusions?	No	Yes
8. Did the authors describe the studies included in enough detail?	Partial Yes	Partial Yes
9. Did the authors use a satisfactory technique for assessing the RoB in studies that were included?	No	Yes
10. Did the authors report the sources of funding for the studies that were included?	Yes	No
11. In case of meta-analysis, did the authors use appropriate methods for the statistical combination of results?	No meta-analysis	Yes
12. In case of meta-analysis, did the authors assess the potential effect of RoB in individual studies on the results of the meta-analysis or other evidence synthesis?	No meta-analysis	Yes
13. Did the authors account for RoB in individual studies when interpreting or discussing the results?	No	Yes
14. Did the review authors provide a reasonable explanation for and discussion of any heterogeneity observed in the results of the review?	Yes	Yes
15. If they performed quantitative synthesis, did the review authors carry out a satisfactory investigation of publication bias (small study bias) and discuss its likely impact on the results of the review?	No meta-analysis	Yes
16. Did the authors report any sources of conflict of interest, including any funding they received for conducting the review?	Yes	No

Study Characteristics

All 11 studies included in the review concluded that PCOS patients have a significantly higher risk of developing NAFLD than apparently healthy controls. Five studies suggested that hyperandrogenism in PCOS patients could be a possible trigger for the development and progression of NAFLD. Most other studies attributed the link to the presence of MetS by assessing factors like body mass index (BMI), waist circumference (WC), homeostasis model assessment of insulin resistance (HOMA-IR), glucose levels, total cholesterol, and triglycerides (TGs). Generally, the studies measured NAFLD by determining the level of hepatic steatosis (HS) or as an increase in aminotransferases. However, Polyzos et al. used non-invasive indices like NAFLD-fat score, lipid accumulation product (LAP), and hepatic steatosis index (HIS) to measure steatosis in 392 study participants.

Additionally, fibrosis-4 (FIB-4), aspartate aminotransferase (AST)-to-Platelet Ratio Index (APRI), BMI-Age-Alanine aminotransferase (ALT)-Triglycerides (BAAT), and BMI AST/ALT Ratio Diabetes (BARD) were used for the measurement of fibrosis [14]. This study concluded that PCOS was more likely associated with HS than hepatic fibrosis. On the other hand, two studies attempted to use transient elastography (TE) to assess NAFLD. Nevertheless, all studies demonstrate a significant risk of NAFLD associated with PCOS. Table 5 shows the types of studies included in this review, and Table 6 summarizes the results of each of the studies in further detail.

**Table 5:** Types of studies included in this systematic review JBI = Joanna Briggs Institute; AMSTAR = Assessment of multiple systematic reviews

Type of Study	Tool Used	Number of Studies
Cross-sectional studies	JBI	7
Case-control studies	JBI	2
Cohort Studies	JBI	1
Systematic Reviews and Meta-analyses	AMSTAR Checklist	1

**Table 6:** Summary of results PCOS = polycystic ovary syndrome; NAFLD = non-alcoholic fatty liver disease; HS = hepatic steatosis; P = p-value; BMI = basal metabolic index; WC = waist circumference; HOMA-IR = homeostasis model assessment-estimated insulin resistance; FAI = free androgen index; HDL = high-density lipoprotein; SHBG = sex hormone binding globulin; MetS = metabolic syndrome; LAP = lipid accumulation product; TG = triglycerides; LSM = liver stiffness measurement; CAP = controlled attenuation parameter; TE = transient elastography; OR = odds ratio; CI = confidence interval; FIB-4 = fibrosis-4; IR = insulin resistance; nmol/L = nanomoles per liter

Author and Year of Publication	Study Type	Purpose of Study	Number of Participants/ studies	Results and Conclusion
Vassilatou et al., 2010 [10]	Case-control study	Investigation of premenopausal PCOS patients (via abdominal ultrasonography and biochemical testing) to determine the presence of NAFLD Assessment of metabolic and hormonal factors correlating with NAFLD and PCOS.	57 premenopausal women with PCOS; 60 age- and weight-matched women without PCOS as controls	36.8% of cases had HS vs 20.0% of controls (P < 0.05) whereas 22.8% of cases had abnormal serum aminotransferases vs only 3.3% of controls (P < 0.01). All participants who had MetS also had evidence of HS. Patients with HS were determined to be 3.55 times more likely to have PCOS [i.e., OR = 3.55 with (95% CI 1.02-5.35)]. Factors that correlate HS with PCOS are: PCOS diagnosis, advanced age, elevated BMI, WC, HOMA-IR, and FAI, as well as decreased HDL and SHBG levels. It was concluded that NAFLD was common in PCOS patients, likely due to increased androgens and metabolic abnormalities.
Jones et al., 2012 [16]	Cross-sectional Case-control study	To determine if PCOS is an independent risk factor for HS and determine if HS is related to hyperandrogenemia	29 PCOS patients and 22 age and BMI-matched healthy control women	Hyperandrogenic women with PCOS were found to have greater liver fat content than non-hyperandrogenic PCOS patients and healthy controls, even after adjusting for BMI, HOMA-IR, and internal and visceral adipose tissue volume. Therefore, it was concluded that hyperandrogenism in PCOS patients, irrespective of the presence of IR and obesity, is associated with HS.
Polyzos et al., 2014 [13]	Cross-sectional study	To investigate the association of non-invasive indices of HS and fibrosis with MetS in PCOS patients versus controls from Greece.	314 PCOS women (77 with MetS and 237 without) and 78 controls	All three steatosis indices were higher in PCOS patients than controls, whereas only two of the four fibrosis indices were higher in PCOS patients. All three steatosis indices were higher in PCOS women with MetS than those without it. Still, only one fibrosis index was higher in PCOS women with MetS. Therefore, the results suggest that indices for steatosis have a greater association with MetS than indices for fibrosis, especially in the PCOS patients
Macut et al., 2016 [15]	Cross-sectional study	To determine the prevalence of NAFLD in PCOS patients from Greece and the most significant risk factors associated with progression to NAFLD in PCOS patients.	600 women with PCOS and 125 BMI-matched healthy women as controls	The prevalence of NAFLD was 50.6% in PCOS patients vs. 34.0% in controls. WC, LAP, insulin and HOMA-IR, total cholesterol, and TGs were higher in PCOS patients than in controls (P < 0.001). NAFLD-liver fat score was most significantly associated with WC, BMI, glucose levels, LAP, HOMA-IR, FAI, and TGs. HOMA-IR and LAP were deemed as independent risk factors for NAFLD in PCOS patients
Kim, JJ et al., 2017 [9]	Case-control study	To analyze the prevalence of NAFLD in non-obese women with or without PCOS and to determine the correlation between NAFLD and PCOS in non-obese Asians	275 non-obese PCOS patients from Seoul and 892 non-obese controls from Seoul	5.5% of the non-obese PCOS patients had NAFLD vs. 2.8% of controls (P = 0.027) after adjustment for age and BMI. Hyperandrogenism in the non-obese PCOS cohort was associated with NAFLD even after adjustment for lipid profile, glycemic status, and IR.
Kumarendran et al., 2018 [22]	Cohort Study	To determine the incidence of NAFLD in PCOS patients and to explore the roles of BMI and hyperandrogenism as risk factors for NAFLD	63 120 women with PCOS selected from a primary care database in the United Kingdom and 121 064 age, BMI, and location-matched controls	The hazard ratio for NAFLD in women with PCOS was 2.23 (95% CI 1.86–2.66, *p* < 0.001), indicating an increased rate of NAFLD in these women. Serum testosterone > 3 nmol/L and SHBG < 30 nmol/L both resulted in increased NAFLD rates. BMI, dysglycemia, and hyperandrogenism contribute to the elevated risk in these patients.
Wu et al., 2018 [28]	Meta-analysis study	To explore the effect of PCOS on NAFLD development and that if the link is direct or due to shared risk factors	17 studies published before May 2017 were included	The OR for NAFLD in PCOS patients was 2.25 (95% CI: 1.95-2.60); therefore, PCOS subjects had a significantly higher risk of developing NAFLD. Prevalence of NAFLD was more common in obese patients vs. non-obese patients. Prevalence of NAFLD in PCOS patients was highest in subjects of Europe, followed by the Asia-Pacific region, and then in America. Hyperandrogenism was deemed as the most influential risk factor, whereas obesity and geography were less influential.
Asfari et al., 2020 [4]	Cross-sectional study	To determine if PCOS is an independent risk factor of NAFLD	77 415 out of 50 785 354 female patients with PCOS, according to National Inpatient Database, from 2002 to 2014	Patients with PCOS had approximately eight times higher odds of having NAFLD even after adjustment for various confounders. PCOS patients were younger and more obese than controls but less likely to have co-morbidities like hypertension, dyslipidemia, and type 2 diabetes mellitus.
Chakraborty et al., 2020 [14]	Cross-sectional study	To investigate the prevalence of HS in young Indian women with PCOS and to determine the efficacy of TE in the assessment of NAFLD	70 Indian women with PCOS and 60 healthy women as controls	The prevalence of HS in women with PCOS was 38.56%, whereas it was 6.67% in controls. The aminotransferase levels were also significantly higher in PCOS patients. Assessment of liver stiffness measure (LSM) and controlled attenuation parameter (CAP) on TE may predict the presence of NAFLD in PCOS patients.
Salva-Pastor et al., 2020 [6]	Cross-sectional study	To investigate the prevalence of NAFLD in Mexican women with PCOS compared to age and BMI-matched controls	49 women of reproductive age with PCOS and 49 healthy women as controls	Prevalence of NAFLD was 69.3% in PCOS patients and 34.6% in controls; (OR=4.26, 95% CI 1.83-9.93). Prevalence of NAFLD was greater in PCOS patients with phenotype A than in other phenotypes. Patients with excess androgens had higher mean CAP on TE than subjects without hyperandrogenism. It was concluded that PCOS serves as an independent risk factor for NAFLD.
Taranto et al., 2020 [12]	Cross-sectional study	To investigate the prevalence of NAFLD in PCOS patients and their associated risk factors, also investigate various indices of HS in these patients	87 Brazilian women with PCOS and 40 controls	NAFLD was discovered in 77% of the PCOS patients compared to 52.5 % of the controls, likely due to elevated serum TGs, alanine aminotransferase, and WC. FIB-4 Index did not correlate with advanced stage of fibrosis, whereas NAFLD score and TE showed some correlation (3.8% and 12% of patients, respectively).

Discussion

PCOS is not simply a disease of the ovaries as some women present without polycystic ovaries on ultrasound as per the name. It is thus considered a multi-organ disease affecting organs like the pancreas, adrenal glands, heart, and liver [29]. In this study, we aimed to understand its consequences in the liver as several studies have suggested an association of PCOS with NAFLD.

PCOS: Independent Risk Factor?

The first case of NAFLD in PCOS patients was discovered in 2005 by Brown et al. in a 24-year-old obese woman with PCOS who underwent investigations for elevated liver enzymes [30]. It was thus speculated that NAFLD might occur in PCOS women since NAFLD and PCOS have common risk factors such as MetS. The research was then directed at confirming the correlation between the two and investigating the primary risk factors involved. Although several studies have suggested that PCOS patients have a higher risk for developing NAFLD, the debate regarding whether PCOS is an independent risk factor for the outcome is ongoing. Vassilatou et al. conducted a study in 2010 amongst 57 Caucasian PCOS patients and 60 Caucasian controls in which they reported that the odds of discovering NAFLD in PCOS patients were 3.55 times higher than in the controls. They also noticed that all subjects with MetS, including the controls, had NAFLD [10]. Therefore, this suggests that MetS may be the main link between the two rather than the diagnosis of PCOS alone.

Other factors like age, obesity, IR, WC, and androgen level also contribute to the development of PCOS. Similarly, Macut et al. reported that HOMA-IR and LAP were the most significant factors involved in the association of NAFLD with PCOS [15]. Based on these two studies, it can be concluded that although the risk for NAFLD is increased in PCOS patients, the reason is not solely the diagnosis of PCOS. Although Vassilatou's study only included 117 subjects and was published more than a decade ago, Macut's study was more recent and included more participants. Nonetheless, their conclusions imply that PCOS is not an independent risk factor for NAFLD.

On the other hand, Asfari et al. conducted a very large cross-sectional study including 77 115 PCOS patients identified from the National Inpatient Sample database from 2002 to 2014. They found that women with PCOS had a four times higher risk of having NAFLD and a lower incidence of hypertension, dyslipidemia, and diabetes mellitus [4]. Since these three conditions are all features of MetS, this implies that MetS may not have such a significant role in the prevalence of NAFLD in PCOS patients as once thought. Additionally, Salva-Pastor et al. came to a similar conclusion while studying Mexican PCOS patients. The study results demonstrated that despite having a lower prevalence of NAFLD in non-obese PCOS patients, PCOS patients still have a higher risk for NAFLD than other women overall [6]. This indicates that while we cannot dismiss the role of obesity in PCOS for developing NAFLD, there are still factors specific to PCOS that lead to NAFLD. Furthermore, they implied that different phenotypes of PCOS have different degrees of risk involved. Interestingly, this study used TE to diagnose NAFLD, whereas most used ultrasonography. Nevertheless, both studies present a strong argument that PCOS is, in fact, independently associated with the prevalence of NAFLD.

Role of Hyperandrogenism in Pathophysiology of NAFLD

Although some studies in the past have argued that PCOS is an independent risk factor for NAFLD, further studies were required to determine which feature of PCOS is the most significant in the progression of NAFLD in these women. In this systematic review, we came across several studies that determined hyperandrogenism in PCOS patients may be the most influential factor in the development of NAFLD. For example, a study conducted in 2012 by Jones et al. presented evidence that hyperandrogenic PCOS women had more liver fat content than PCOS women with normal androgens or women who served as controls [16]. This observation was held even after adjusting for BMI, HOMA-IR, and adipose tissue volume in the internal and visceral organs. This suggests that hyperandrogenic PCOS women can develop NAFLD despite having normal weight and insulin sensitivity. However, this study only included 51 participants, whereas Kim et al. demonstrated the same observation in 2017 with 1167 participants. Hyperandrogenism, signified by elevated free testosterone and free androgen index (FAI), was credited as a significant individual risk factor for NAFLD in the 275 non-obese PCOS patients studied compared to 892 non-obese healthy controls [9]. This conclusion seems plausible as some studies have suggested that the level of androgens and their receptors play an important role in lipid metabolism in the liver [9]. The results of this study were significant because previous studies on the subject had only discussed the prevalence of NAFLD in obese PCOS patients, therefore connecting the association to elements of MetS like obesity, dyslipidemia, and IR. However, it is important to note that this study used ultrasonography to assess NAFLD instead of liver biopsy (the gold standard for diagnosis), although they argued that it was cost-effective, sufficiently sensitive, and specific for a study of this scale.

In 2018, Kumarendran et al. decided to further explore the idea of hyperandrogenism as the driver behind NAFLD in PCOS patients. They conducted a wide-scale retrospective cohort study involving 1,84,184 participants selected from a general practice electronic database in the United Kingdom called The Health Improvement Network (THIN). The study found that among the 63,000 PCOS patients, there was a two-fold greater risk of NAFLD development than in the controls [22]. Additionally, they argued that although the reason for NAFLD development in this cohort may be a complex interplay between obesity, IR, and hyperandrogenism, hyperandrogenism played a more significant role. They attempted to solidify this argument by measuring NAFLD risk in two independent cohorts that did not have PCOS. They discovered that women with serum testosterone levels greater than three nmol/L and sex hormone binding globulin (SHBG) less than 30 nmol/L had an increased rate of NAFLD occurrence [22].

Nonetheless, one major shortcoming in this study was minimal documentation of criteria used to diagnose PCOS and NAFLD. This is concerning because it could mean that the two independent cohorts which demonstrated an increased risk of NAFLD in hyperandrogenic women could be women who had PCOS but were inadequately diagnosed. Additionally, a meta-analysis conducted by Wu et al. in 2018 suggested that hyperandrogenism was a major risk factor irrespective of BMI and geographic differences in the selected study population [28]. Their reasoning behind this argument was that hyperandrogenism could lead to NAFLD directly by promoting visceral fat deposition or indirectly by promoting IR and an inflammatory state [28]. Despite the few studies included in the meta-analysis, the idea that hyperandrogenism is the most significant factor involved in the early development of NAFLD persists.

Extent of NAFLD

Most of the studies included in this systematic review measured NAFLD using ultrasonography despite acknowledging that it is inferior to liver biopsy. Although understandably, performing a liver biopsy on each participant in a study is unfeasible, perhaps another method exists which will not only diagnose but also stage and monitor the extent of liver disease. For example, Polyzos et al., 2014, attempted to use non-invasive indices to measure steatosis and fibrosis, representing different stages in liver disease. They discovered that the indices used to measure HS were significantly higher in PCOS patients, especially those with MetS, whereas fibrosis indices correlated poorly with PCOS [13]. Therefore, this suggests that PCOS may only lead to a mild, reversible form of liver disease rather than a much more severe form like cirrhosis. It may even suggest that these patients' natural history of liver failure is prolonged. However, the extent of liver disease in PCOS patients and the frequency and speed of progression to cirrhosis are areas of research that need further exploration.

Another method used to diagnose NAFLD in some studies is TE. This non-invasive imaging technique measures liver fibrosis by determining liver stiffness measurement (LSM) and controlled attenuation parameter (CAP). Chakraborty et al. in 2020, for instance, set out to determine TE's effectiveness in assessing NAFLD in a cohort of Indian women with PCOS. They discovered that both LSM and CAP were higher in PCOS women, but only CAP significantly correlated with the other measures of liver fat content like liver fat score (LFS) and HIS [14]. Because LSM values indicate liver fibrosis and CAP is a measure of HS, this reinforces the idea that PCOS may only lead to a mild NALFD.

Similarly, Taranto et al. also used TE and other non-invasive indices to diagnose and stage NAFLD in a subset of Brazilian women with PCOS. Like, Chakraborty et al., they found strong evidence for the correlation but were unable to find a strong correlation between PCOS and liver fibrosis [12]. Thus, it can be argued that TE may be a more favorable alternative for NAFLD measurement than ultrasonography; however, further study is still warranted.

Strengths and limitations

Strengths of this systematic review include the utilization of studies that contain subjects from different countries like Greece, Brazil, India, South Korea, Mexico, and the United Kingdom. This allows the comparison of results among different populations and enhances the significance of the results. Additionally, various qualitative studies were included with varying sample sizes to analyze the significance of the data. On the other hand, limitations include a lack of access to a few relevant full-text papers despite an attempt to contact authors. Additionally, the limited number of databases searched further decreases the number of valuable articles that could have contributed to enhancing the systematic review.

## Conclusions

In conclusion, our systematic review aimed to determine whether PCOS increases the risk of developing NAFLD. The results of the reviewed studies have demonstrated an increased risk of NAFLD in PCOS patients of reproductive age in various countries. Despite the significance of risk factors like obesity and IR in developing NAFLD, hyperandrogenism seems to be the most influential factor in PCOS patients. Therefore, early recognition is warranted to control and potentially reverse liver disease as most cases are limited to HS. The younger age of the patients may explain this. However, the natural history of NAFLD in PCOS patients requires further study. Furthermore, TE may be used as an alternative for ultrasonography to diagnose better and monitor this population's natural history of NAFLD.
